# Translating Psychedelic Therapies From Clinical Trials to Community Clinics: Building Bridges and Addressing Potential Challenges Ahead

**DOI:** 10.3389/fpsyt.2021.737738

**Published:** 2021-11-04

**Authors:** Martin L. Williams, Diana Korevaar, Renee Harvey, Paul B. Fitzgerald, Paul Liknaitzky, Sean O'Carroll, Prashanth Puspanathan, Margaret Ross, Nigel Strauss, James Bennett-Levy

**Affiliations:** ^1^Turner Institute, School of Psychological Sciences, Monash University, Melbourne, VIC, Australia; ^2^Psychedelic Research in Science & Medicine (PRISM) Ltd, Melbourne, VIC, Australia; ^3^Department of Psychosocial Cancer Care & Palliative Care, St Vincent's Hospital Melbourne, Melbourne, VIC, Australia; ^4^Centre for Mental Health, Department of Psychological Sciences, Swinburne University of Technology, Melbourne, VIC, Australia; ^5^Millswyn Clinic, Melbourne, VIC, Australia; ^6^Epworth Centre for Innovation in Mental Health, Epworth Healthcare, Melbourne, VIC, Australia; ^7^Department of Psychiatry, Monash University, Melbourne, VIC, Australia; ^8^Department of Psychiatry, School of Clinical Sciences, Monash University, Melbourne, VIC, Australia; ^9^Wild Mind Institute, Melbourne, VIC, Australia; ^10^Enosis Therapeutics, Melbourne, VIC, Australia; ^11^Medicine, Dentistry and Health Sciences, University of Melbourne, Melbourne, VIC, Australia; ^12^Centre for Mental Health, Swinburne University, Melbourne, VIC, Australia; ^13^University Centre for Rural Health, The University of Sydney, Lismore, NSW, Australia

**Keywords:** psychedelics, mental health, clinical research, translation, community clinics, psilocybin, trauma, depression

## Abstract

Research exploring the potential of psychedelic-assisted therapies to treat a range of mental illnesses is flourishing, after the problematic sociopolitical history of psychedelics led to the shutdown of clinical research for almost 40 years. Encouraged by positive results, clinicians and patients are now hopeful that further interruptions to research will be avoided, so that the early promise of these therapies might be fulfilled. At this early stage of renewed interest, researchers are understandably focusing more on clinical trials to investigate safety and efficacy, than on longer-term goals such as progression to community practice. Looking to identify and avoid potential pitfalls on the path to community clinics, the authors, a group of Australian clinicians and researchers, met to discuss possible obstacles. Five broad categories of challenge were identified: 1) inherent risks; 2) poor clinical practice; 3) inadequate infrastructure; 4) problematic perceptions; and 5) divisive relationships and fractionation of the field. Our analysis led us to propose some strategies, including public sector support of research and training to establish best practice and optimize translation, and funding to address issues of equitable access to treatment. Above all, we believe that strategic planning and professional cohesion will be crucial for success. Accordingly, our key recommendation is the establishment of a multidisciplinary advisory body, broadly endorsed and representing all major stakeholders, to guide policy and implementation of psychedelic-assisted therapies in Australia. Although these challenges and strategies are framed within the Australian context, we sense that they may generalize to other parts of the world. Wherever they apply, we believe that anticipation of potential difficulties, and creative responses to address them, will be important to avoid roadblocks in the future and keep the “psychedelic renaissance” on track.

## Introduction

Half a century after a range of sociopolitical factors rendered psychedelic clinical research and practice untenable, a widely touted “psychedelic renaissance” is well-underway (1, 2). Research has accelerated to the extent that psychedelic science is now one of the fastest-growing disciplines in medical research (3).

Reflecting the primary role of research in medical innovation, much of the recent literature on psychedelic-assisted therapies (PAT) has focused on clinical trials (4). In this paper, we define PAT as the administration of classic psychedelic drugs - and empathogens such as MDMA – in combination with psychotherapeutic intervention administered by appropriately trained and accredited clinicians for the treatment of a range of mood disorders and mental health issues including anxiety, depression post-traumatic stress disorder, substance use disorders and a range of obsessive-compulsive disorders. Recognizing that the next step toward implementation of innovative approaches is translation, we look beyond research to anticipate potential issues and possible solutions in moving from clinical trials to community clinics.

Two contextual factors have emerged to frame our analysis. The first is that psychedelics are “disruptive psychopharmacologies” (5), often having powerful impacts beyond those of any pharmacological agents in current use. They can open an individual to strong emotions - among them fear, rage, joy, sorrow, and shame - commonly experienced during psychedelic therapy sessions, along with vivid visual images, deep memories, and powerful insights. Indeed, the phenomenological and emotional effects of psychedelics appear central to the therapeutic process (6–8).

A second consideration is that community and media interest in PAT are unlike anything we have seen before in psychopharmacology or psychotherapy. In the face of worsening mental health statistics, and given a promising therapeutic modality that has been outlawed for almost fifty years, there is strong pressure from some advocacy groups to fast-track, or even bypass, clinical research and rapidly implement PAT in community settings.

However, undue haste in translation to community clinics could compromise essential aspects of efficacy, safety, and equity, ultimately threatening the sustainability of PAT. Issues ranging from training and accreditation to regulation and economics are all emerging as the approach is being explored anew.

Given these concerns, it is critical to avoid the pitfalls of the past. Thus, the authors of this paper - all involved as researchers, trainers, or clinicians in Australian clinical trials of psychedelic-assisted psychotherapy - met to consider possible pathways from clinical trials to community clinics, which are defined in this paper as mental health clinics outside large hospitals and other institutions, and generally represent the first line of treatment in the local community setting.

We identified five broad categories of challenge, encompassing 1) inherent risks; 2) potential for poor clinical practice; 3) issues surrounding training and infrastructure; 4) problematic positions; and 5) professional and therapeutic relationships, and the potential for divisiveness and fractionation.

We proceed in this Perspective to frame and elaborate on those challenges, then recommend some strategies to address them in hopes of navigating a smooth path ahead. Although we approach the subject from an Australian perspective, we expect that some of our observations, conclusions and recommendations may apply to other contexts worldwide, notably in the USA, Canada, Europe and Israel, as the potential of psychedelic therapies continues to be explored (9, 10).

### Inherent Risks Associated With Treatment

Renewed research is lending support to findings from historical studies that psychedelics do not pose significant risk when administered to suitable individuals with due care, at therapeutic doses, in clinical settings (4). However, careful screening and clinical care are key to minimizing the possibility of adverse events and negative outcomes (11). If requisite measures are not taken, patients may be exposed to undue personal risk.

#### Medical Risks

Adverse physiological effects of psychedelics are rare, relating mainly to cardiovascular and other peripheral responses to their serotonergic and adrenergic actions. All are transient and, given appropriate precautions, no serious adverse events have been recorded in recent clinical trials of PAT (12, 13).

More significant risks are posed by interactions between psychedelics and concomitant medications. A comprehensive list of medications that pose significant medical risks to participants in clinical trials has been compiled over time (11, 14). Lack of adherence to these guidelines in community clinics, due for example to lack of appropriate training, could have significant health consequences for patients.

#### Psychological Risks

Psychological risks (15–17) include susceptibility to psychotic or manic episodes, trauma associated with difficult experiences, and rebound reactions of depression or anxiety. Negative emotional responses generally resolve with appropriate preparation and post-session support. When these effects persist, however, intervention may be required to address them and minimize risk of more serious consequences such as despair, existential crisis, and self-harm.

Negative outcomes such as delusions and other manifestations of incipient psychosis, though rare, also constitute serious conditions that could deteriorate without active intervention in the community setting.

### Potential for Poor Clinical Practice

Successful translation of PAT to community settings will depend on adequate expertise, procedures, and ethical standards (12). The potential for poor clinical practice is significant, as are the consequences. Ultimately, poor practice could prove to be the greatest hurdle to successful translation of PAT.

#### Translation From Research

Research protocols may not translate well to community clinics (12, 18, 19), which can face particular operational challenges including inconsistent referrals, high patient throughput, limited scope for follow-up, and cost pressures (20).

In clinical trials, risks are minimized using strict exclusion criteria to screen for a range of pre-existing medical and psychiatric conditions. Such careful measures may not survive translation, due for example to lack of skills and training, or financial constraints.

Manualized or otherwise narrowly specified research methods may translate poorly to clinical settings, where the therapeutic needs of individual patients can vary markedly. Comorbid conditions may be underestimated or overlooked. Misdiagnosis could lead to unnecessary treatment, as could unjustified off-label use, or enthusiastic but inappropriate administration of PAT. Finally, handover back to a referring doctor, or any form of ongoing care, may not take place following treatment.

Consequences in any of these situations could be grave, extending for example to risks of medical emergency, self-harm, or even suicide, in cases of inadequate screening for comorbid physiological or psychiatric conditions, inappropriate application of psychedelic therapies, or relapse of long-term, treatment-resistant mood disorders such as depression or post-traumatic stress.

#### Regulatory, Medical, and Market Forces

Crucial elements of PAT risk being undermined by various factors other than clinical capability. Aspects central to clinical efficacy may be compromised to fit the current norms of Western medicine, such as directive interactions, professional distance, short consultation times, manualized treatment, and the dominant pharmacotherapeutic model. Similar concerns apply to financial considerations, including pressures to maximize profits, hyped marketing, and dependent consumers.

#### Inadequate Training

It is commonly noted - particularly by skeptics of PAT - that research is conducted primarily by clinicians who are particularly personally committed to the approach. Additionally, they are carefully trained to support acute altered states of consciousness, and to guide trial participants through the critical phases of preparation and integration.

Scaled delivery of psychedelic psychotherapy to the community may prove difficult if training and accreditation are inappropriate or insufficient to meet demand – as is the case with other modalities (20). Key questions include appropriate candidates, capacity of training providers, educational content, certification, and ongoing regulation.

#### Boundary Violation

Transgression of ethical boundaries is an ever-present risk for clinicians and subjects when working with altered states of consciousness, given therapist-patient power imbalances and potentially traumatic states in vulnerable patients (21–24). Research protocols specify male-female therapist dyads, ongoing supervision by experienced therapists, transparent practices, and ultimately oversight by Ethics Committees and Review Boards.

Maintaining appropriate standards of treatment and ethical practice in community settings may be problematic unless independent regulatory and supervisory processes can be assured (25).

### Infrastructure Issues

#### Access to Treatment

PAT is labor-intensive during the clinical intervention, typically requiring 30–50 h of joint input by two appropriately trained and accredited clinicians (26). While the short-term costs are high - particularly if psychiatrists or specialist physicians are providing care for extended periods - they may be justified if therapeutic benefits are sustained and produce substantial functional improvements.

We are deeply concerned, however, that PAT will be inaccessible to many of the most vulnerable and in-need patient populations – including Australia's First Nations people, whose deep history and culture we respectfully acknowledge, and in whom we recognize the disproportionate incidence of intergenerational trauma-related illness experienced by First Nations peoples worldwide (27).

#### Training and Accreditation

Australia currently faces a shortage of mental health practitioners that will probably impact the provision of PAT. Very limited training options currently exist, so development of training to meet anticipated demand for appropriately skilled psychedelic psychotherapists and supervisors will present challenges over the coming decade.

#### Setting

One robust finding from decades of PAT research is the importance of clinical setting for positive therapeutic outcomes (26, 28, 29). Efficacy of PAT can be compromised by a typical clinical environment, so modification of existing clinics will be required to render them fit for purpose. Substantial capital investment also may be needed to build new clinical facilities designed specifically for effective PAT. This may place significant cost pressures on community provision of PAT.

### Problematic Perceptions, Positions and Expectations

#### Perceptions

Global media and social commentary relating to psychedelics until recently followed conservative lines, either promulgating a pathogenic narrative and highlighting their perceived dangers (30), or simply dismissing their potential for therapeutic use. Thus, for some 40 years the main challenges to both clinical research and translation of PAT were based on problematic perceptions.

#### Positions and Expectations

Contemporary perceptions have changed dramatically, however, and it is now increasingly difficult to find serious opposition to PAT. It appears instead that a greater threat may be posed by enthusiastic proponents of accelerated, even immediate, approval for medical practitioners to prescribe psychedelics and administer PAT. Australian media are embracing this narrative, cultivating positive expectations of PAT in a community beset by mental illness and convinced that current treatments are not only ineffective, but may be exacerbating harm.

Advocacy is currently focused on Australia's Special Access Scheme, to enable prescribers and therapists to administer PAT in the community setting, outside the normal channels of medicines approval by the Therapeutic Goods Administration (TGA) (31). A significant concern is that this could occur with minimal training or accreditation, supervision or mentorship, and no independent oversight - with potentially negative outcomes (32).

Nevertheless, several more years of clinical research prior to rollout – even assuming positive trial outcomes and smooth approval processes – may not sit well with a community struggling with mental illness. Frustration and pent-up demand for effective therapies are expressed through disappointment, even rage, upon exclusion from clinical trials based on criteria that some clearly regard as being overly conservative. Countering this is a strong argument that research needs to be completed (33), and many therapists will need to be trained before community needs, let alone expectations, are met.

### Divisive Relationships and Fractionated Field

Public discussion is divided and emotionally charged. Differences have already emerged between those pushing for rapid regulatory change and clinical rollout based on limited, even anecdotal, evidence, and those who favor a more measured approach ultimately informed by research (33). Another division has emerged between the psychotherapeutic and pharmacotherapeutic models of mental health treatment. In this emotive environment, an otherwise healthy diversity of opinions can escalate into destructive divisions among interested groups.

#### Fractionated Field

Professional organizations have only recently acknowledged the potential of psychedelic therapies after a long silence. Now that it appears to offer a viable alternative to established approaches, we sense that tensions may emerge among the mental health professions as to who might be best suited to deliver PAT. Effective translation of PAT to community clinics may be jeopardized if professional divisions prove resistant to collaboration and consultation.

#### Commercial Interests

If PAT is rolled out to community clinics over the coming decade, competition will likely emerge between public health and corporate interests in the sector. Such a division could favor patients who are better placed to pay. To balance this disparity, support of psychedelic therapies through the Pharmaceutical Benefits Scheme (PBS) and Medicare Benefits Schedule (MBS) in Australia (34) will be crucial for equitable access through the community health network.

Meanwhile, the entry of for-profit interests from more mature commercial environments such as Canada and the USA has already commenced (35). Commercial interests seek to control the supply of psychedelics for research, and there are indications that future access to medicinal psychedelics by community clinics may be contractually bound to global enterprises. Ultimately, our concern is the compromise of patient priorities, especially efficacy, safety, and equity, in favor of commercial considerations (36). The first step toward ensuring a patient-centered future is a commitment to Open Science and transparent translation pathways (3).

### Discussion and Recommendations

Broad-scale rollout of PAT from research to community clinics in Australia is conceivable over the coming decade, and the potential impact of such a move cannot be overstated. However, ensuring adequate expertise, protocols and standards of care while scaling to community settings may be challenging. Hence, the process will require careful planning and navigation to avoid a range of issues.

Our key recommendation is establishment of a multidisciplinary **Australian Advisory Committee for Psychedelic Therapies**, representing research, clinical, regulatory, industry, and community interests. Such a peak body would provide guidance to government, professional organizations, and other stakeholders in training and accreditation, infrastructure development, community education, and regulatory matters. It could also provide guidance on the most appropriate ways to invest in the critical area of translation, to ensure adequate focus on the ultimate goal of successful community access to gold-standard PAT.

Our recommendation is inspired by the recent proposal of a National Advisory Council and subsidiary Credentialing Council to undertake strategic oversight of psychedelic therapies in Canada (37). In the Canadian model, those councils would advise on, *inter alia*, ethical codes of conduct, education and training, core clinical competencies, and accreditation of clinicians and practitioners to provide psychedelic-assisted therapies to clients.

We see very similar issues and challenges facing the Australian clinical community to those identified in Canada by Rochester and colleagues (37). Based on these similarities, we see merit in recommending an Advisory Committee that draws upon the expertise of the medical colleges and professional associations, alongside academic institutions, cultural groups, regulators, and the broader community. Importantly, consultation must be undertaken with Australia's First Nations people to establish if, how, when, and in what contexts PAT might be offered to Australia's Indigenous communities in a collective effort to heal deep historical traumas (27, 38). Little or no record of any traditional use of psychedelics by Australia's Indigenous people – which would constitute deep knowledge that rightly remains with those communities – is available to the majority of researchers, clinicians and regulators. Thus, we are sensitive to the cultural implications of this recommendation and hope for a respectful consultative process to ensure the best possible outcomes for all Australians.

We now discuss how such an Advisory Committee could contribute in several key areas to optimize the translation of PAT to the community setting.

#### Training and Accreditation

Training and accreditation of a large cohort of psychedelic therapists will be essential to meet anticipated community demand for PAT. Regardless of formal qualifications and prior clinical experience, specialized yet non-hierarchical training and accreditation in PAT will be important for safety, efficacy, and sustainability. Consequently, all practitioners should be qualified to administer PAT only after completion of comprehensive psychedelic therapist training and accreditation.

Therapist training ideally should be incorporated into teaching curricula at universities and hospitals. For now, most trainee therapists will be instructed by clinicians working in clinical research - an approach likely to achieve the greatest acceptance of PAT among the medical community. We see this evolving into broader-scale therapist education within the institutions that have fostered psychedelic research, by trainers who themselves have gained their knowledge and skills through that research framework.

One factor specific to clinical practice is that for the foreseeable future, psychedelic medications, if approved for clinical use, are most likely to be prescribed by specialists such as psychiatrists and addiction physicians. However, the psychotherapeutic needs of PAT may not be met by those practitioners without specialized training, so one solution could be to define two levels of psychedelic-relevant training for psychiatrists – one for PAT therapists and the other for PAT prescribers. Such a system exists in other countries (39). Alternatively, licensing to prescribe psychedelics could be extended to other clinicians such as physicians, palliative care specialists, and general practitioners, contingent on suitable accreditation.

Above all, training programs and clinicians themselves should be accredited through a dedicated professional association to maintain safety and standards in the translation to community clinics. We recommend the foundation of a dedicated cross-disciplinary professional body to oversee the field, given the broad range of backgrounds from which psychedelic therapists will be drawn. The Canadian model of a Credentialing Council (37) would provide a good starting point for our proposed Accreditation Committee, which we see taking a supporting role to that of the Advisory Committee on Psychedelic Therapies.

#### Communication and Consultation

Communication and consultation among clinicians, researchers, regulators, and other stakeholders will minimize delays and optimize outcomes in the rollout of PAT to community clinics. Our sense is that the professional community, not to mention people who might benefit from PAT, will be best served by cohesion and common purpose. This will be achieved by coordinated communication and consultation among sectors of the professional community, and greater engagement between mental health professionals and the community.

We see the Advisory Committee being a central node of communication among a broad range of stakeholders, from health professionals and researchers to regulators, cultural representatives, and the community.

#### Regulatory Oversight

Regulatory oversight applies through the established processes of drug scheduling, medicines approval, and healthcare regulation that have been in place in their present form for some 60 years, and essentially have guided our clinical use of drugs to maximize benefits while minimizing harms.

While regulation is necessary to ensure safety and ethical practice, we recommend broad consultation with the community and, in return, broad community support for regulatory measures to ensure safety and equity of access to PAT (40).

The proposed Advisory Committee would play an important role in advising government and statutory bodies, along with professional organizations and representatives of the legal profession, to administer PAT in the community setting for maximum benefit at minimum cost.

#### Financial Considerations

Financial considerations underpin several themes and associated recommendations of this paper. Public funding of evidence-based interventions is needed to ensure universal affordability and accessibility, and evidence is building for the safety and efficacy of PAT. We strongly support funding of PAT within the public health system, reinforced by a robust regulatory environment to deal equitably with commercial aspects – including issues related to intellectual property (41) - of psychedelics and the provision of PAT. Accordingly, our recommendation is for the translation of PAT to community clinics to be supported by the public health system in Australia, based on economic analysis of potential cost savings to the health system in the long term.

We also recommend government funding of clinical trials based on statistically meaningful numbers and long-term follow-up, including post-approval monitoring, to evaluate safety and sustainability of therapeutic outcomes (42). This appears imminent in Australia, through the extension of the Federal Government's competitive grant scheme, the Medical Research Future Fund (43, 44) specifically to fund psychedelic medical research.

Ultimately, we see the proposed Advisory Committee including expertise in health economics and the social sciences, to provide financial and social perspectives on successful translation of PAT to the community setting.

## Conclusion

The successful translation of PAT from clinical trials to community clinics is not guaranteed to be smooth and free from challenges. While we are cognizant of the limitations of our Perspective, which largely reflect the small size and professional orientation of our working group toward research and clinical practice, and the fact that our analysis focuses primarily on the current Australian situation, we have endeavored to identify some of those challenges and offer some solutions that we expect to be appropriate in Australia and many other parts of the world.

We believe our key recommendation, the establishment of an Australian Advisory Committee on Psychedelic Therapies and a supporting Accreditation Committee, will be a critical step toward the successful translation of PAT from research to community clinics.

We are heartened by the active and constructive conversation that has emerged within the public discourse in recent years. Hitherto, the Australian Government and professional organizations such as the Royal Australian and New Zealand College of Psychiatrists (RANZCP), the Australian Psychological Society (APS), and the Australian Medical Association (AMA), have been, at best, silent on the subject. Recently, all have started to engage in respectful and positive consideration of the potential being shown by these game-changing, even disruptive, therapeutic approaches. We are encouraged by the apparent willingness of these stakeholders, along with increasing numbers among the broader community, to consider the potential of psychedelic-assisted therapies after many years of exclusion and neglect.

## Data Availability Statement

The original contributions presented in the study are included in the article/supplementary material, further inquiries can be directed to the corresponding author/s.

## Author Contributions

JB-L: proposed the rationale, convened the in-person and online meetings among co-authors, reviewed literature, and contributed substantially to the drafting and editing of the paper. MW: contributed ideas, collated group input, reviewed literature, composed the paper, edited all drafts, and completed and submitted the paper. DK: contributed ideas and substantial input to the drafting and editing of the paper. RH, PF, PL, SO'C, PP, MR, and NS: contributed ideas and editorial input. All authors reviewed and approved the submitted version.

## Conflict of Interest

MW is Executive Director of Psychedelic Research in Science & Medicine PRISM Ltd, an Australian DGR-1 health promotion charity supporting psychedelic medical research. PF has received equipment for research from MagVenture A/S, Nexstim, Neuronetics and Brainsway Ltd; has received funding for research from Neuronetics; and is a founder of TMS Clinics Australia. PL is a member of the Medical Advisory Board of Incannex Healthcare Ltd. The remaining authors declare that the research was conducted in the absence of any commercial or financial relationships that could be construed as a potential conflict of Interest.

## Publisher's Note

All claims expressed in this article are solely those of the authors and do not necessarily represent those of their affiliated organizations, or those of the publisher, the editors and the reviewers. Any product that may be evaluated in this article, or claim that may be made by its manufacturer, is not guaranteed or endorsed by the publisher.
